# TV, computer, tablet and smartphone use and autism spectrum disorder risk in early childhood: a nationally-representative study

**DOI:** 10.1186/s12889-022-13296-5

**Published:** 2022-04-30

**Authors:** Maria Melchior, Katharine Barry, David Cohen, Sabine Plancoulaine, Jonathan Y. Bernard, Karen Milcent, Malamine Gassama, Ramchandar Gomajee, Marie-Aline Charles

**Affiliations:** 1 Sorbonne Université, Inserm, Institut Pierre Louis d’Epidémiologie Et de Santé Publique (IPLESP), Paris, France; 2 Department of Child and Adolescent Psychiatry, Pitié-Salpêtrière Hospital, Paris, France; 3 CNRS UMR 7222, Institute for Intelligent Systems and Robotics, Sorbonne University, Paris, France; 4 Centre for Research in Epidemiology and StatisticS (CRESS), Université de Paris, Inserm, Inrae, 16 Av Paul Vaillant-Couturier, 75004 Paris, France; 5 Agency for Science, Technology and Research (A*STAR), Singapore Institute for Clinical Sciences (SICS), Singapore, Singapore; 6 UMS Elfe Team, Ined, Inserm, EFS, Aubervilliers, France

**Keywords:** Screen media, Autism spectrum disorder, Birth cohort study, ELFE

## Abstract

**Background:**

Screen media use in early childhood has largely increased in recent years, even more so during the COVID-19 epidemic, and there is much discussion regarding its influence on neurodevelopment, including Autism Spectrum Disorder (ASD).

**Methods:**

We examined the relationship between use of TV, computer, tablet and smartphone at age 2 years and risk of ASD assessed in telephone-based questionnaires among 12,950 children participating in the nationally representative ELFE (‘Etude Longitudinale Française sur les Enfants’) birth cohort study in France.

**Results:**

In inverse-probability weighted (IPW) multinomial regression analyses, children’s weekly or daily screen media use was associated with an increased likelihood of an intermediate risk of ASD (IPW-controlled OR for weekly use:1.07, 95% CI 1.02—1.12; IPW-controlled OR for daily use:1.05, 95% CI 1.02—1.08) but inversely associated with a high risk (IPW-controlled OR for weekly use: 0.60, 95% CI 0.50—0.73; IPW-controlled OR for daily use: 0.75, 95% CI 0.62—0.91), as ascertained by the M-CHAT. This was confirmed when studying TV as well as computer/tablet exposure separately.

**Conclusions:**

Overall, our nationally-representative study conducted among a large sample of 2-year-old children, indicates a complex relationship between screen exposure and ASD risk.

## Background

While children’s use of screen-based media is ubiquitous across high-income countries, and seems to have increased since the beginning of the COVID-19 pandemic [1], growing evidence suggests that exposure in infancy and early childhood is associated with unfavourable developmental outcomes over the short and mid-term [2–5]. In particular, there has been suggestion that high levels of screen-based media viewing are related to a higher level of risk of autism-spectrum disorder (ASD) [2, 6].

Although genetics play an important role with regard to the risk of ASD and symptoms of neurodevelopmental delay, evidence suggests that certain environmental exposures could also be involved [7]. Media screen exposure is a candidate, as children who early on in life spend a lot of time watching screens – particularly if their content is poor and/or if they are unsupervised—are less likely to play, be physically active, and engage in interactions with other children and adults, all of which could be related to worse neurodevelopmental outcomes [3, 5]. However, while it has been documented that children and adolescents diagnosed with ASD are more exposed to screens [2], research examining whether early screen exposure precedes later ASD diagnosis has been limited. A recent study based on 2,100 US-based children participating in the nationally representative National Children’s Study (NCS), found no association between exposure to TV/DVD watching at 12 and 18 months and a high risk of ASD at age 2 years, although the authors did report a linear association with the number of ASD symptoms [8]. To our knowledge, associations between the use of other forms of screen media, including computers/tablets and smartphones and children’s risk of ASD, have not yet been evaluated.

Our aim was to evaluate the relationship between use of TV, computer/tablet and smartphone in relation to neurodevelopmental difficulties and particularly ASD risk in early childhood, in a general population setting.

## Methods

Data for this study come from the ELFE (“Étude Longitudinale Française depuis l'Enfance”) study, a nationally representative birth cohort of 18,329 children born in France in 2011 in 320 maternity wards selected using random sampling methods, with the aims of studying children’s developmental and health trajectories through adulthood [9]. Inclusion criteria were: singleton or twin birth at ≥ 33 weeks of gestation, mother of ≥ 18 years of age and not planning to move outside of Metropolitan France in the three years following study inclusion. Mothers had to be able to give consent either in French, English, Arabic or Turkish. Briefly, participating mothers were invited to take part in the study and completed a research questionnaire after delivery during their maternity stay, then mothers and fathers were contacted again for a telephone interview when the child was aged 2 months, 1 year and 2 years.

In the 2-year ELFE study telephone interview, parents reported on their child’s TV watching, use of a computer/tablet and smartphone. If the child watched television or used a computer/tablet, parents were additionally asked the number of hours during weekdays and weekends, based on which we estimated average exposure. Frequency of use of television and computers/tablets being very distinct, we estimated the level of exposure in hours per day and per week, respectively.

Risk of neurodevelopmental difficulties at 2 years was assessed using the Modified Checklist for Autism in Toddlers (M-CHAT), a validated 23-item screening instrument [10, 11]. We used the recommended cut-offs to define risk of ASD: low (0–2), intermediate (3–6), and high (7–23) risk.

Because children who use screens and those who do not are likely to different with regard to many different characteristics, covariates were controlled for using propensity scores, which were calculated based on the variables listed below [12]:

1. *Family socio-demographic characteristics* reported by the mother after delivery: maternal age (< 30 vs. ≥ 30 years – the average age at first childbirth for women), paternal age (< 30 vs. ≥ 30 years), maternal and paternal birth place (France vs. Other), mother’s educational level, number of children living in the household (1, 2–3, ≥ 4), maternal and paternal residential area (Greater Paris Region; Paris and suburbs, North-West, East, West, South-West, Centre-East, and South-East) and household income per capita assessed when the child was 2 years (divided into 3 categories based on the minimum wage, average wage and above average wage in 2013, https://www.insee.fr/fr/statistiques/1375188) (< 1,423; 1,423–1,996; > 1,996 euros/month).
2. *Maternal mental health* reported by the mother when the child was 2 months old (Edinburgh Postnatal Depression Scale (EPDS) – a score of ≥ 12 being considered as indicative of possible depression, yes vs. no [13]).
3. *Pregnancy and child characteristics* collected from the child's medical records: child sex (male vs. female), age at the time of assessment (< = 24, 25, 26, or > 26 months), season of birth (winter; spring; summer; autumn), prematurity (< 36 vs ≥ 37 weeks of gestation), if the child was a twin (yes vs. no), weight for gestational age (based on a z-score and categorized as small (< 10^th^ percentile), average (10-90the percentile), or large (> 90 percentile)), and Apgar score at 5 min (alarming/fair vs. good).
4. *Postnatal characteristics* reported by the mother at age 2 years: childcare arrangements (parents or family member, centre-based childcare, nanny or childcare professional) and the number of early stimulation and development activities (ex. painting/drawing, storytelling, singing, reading, doing puzzles) the child completed with his/her parents in the preceding month (≤ 4, 5–6, 7–9, >  = 9 activities).

### Statistical analyses

Using survey-weighted data, which takes into account the initial sampling plan and baseline participation as well as longitudinal attrition rates to produce nationally representative estimates for children born in 2011 (https://www.elfe-france.fr/fichier/rte/178/Coté%20recherche/Weighting-Elfe-surveys-general-document.pdf), we assessed associations between screen media use and children’s neurodevelopmental risk (high, intermediate vs. low as reference) at age 2 years. Missing covariate data (maximum 3.1%) were imputed 10 times using fully conditional specification (MI-FCS). To balance differences between screen exposure groups, propensity score weights were estimated with a generalized boosting model (GBM) using Kolmogorov–Smirnov (KS) means. Then, multinomial logistic regression models weighted on the product of Inverse Probability Weights (IPW) and survey weights, yielded Odds Ratios (ORs) with the associated 95% Confidence interval (95% CI) and a *P* < 0.05 examining statistical significance [14]. Missing covariate data (maximum 3.1%) were imputed 10 times with fully conditional specification (MI-FCS). All data were analysed using R 4.0.3 and SAS V9.4.

## Results

A total of 12,950 children were included in the present analysis (Table 1), with 12,012 children with available information on weekly TV, computer or tablet use, and respectively 11,609 and 12,498 children with available information on the frequency of TV and computer/tablet use. Respectively 29.9 and 1.1% of children were classified as having an intermediate and high risk of neurodevelopmental difficulties. 66.4% of children used screen media daily, mostly TV (63.3%, 9.8% being exposed > 2 h/day); 30.5% used a computer/tablet weekly and 26.7% used a smartphone. As shown in Table 1, all studied covariates were associated with children’s screen media use.

**Table 1:** Description of family socio-demographic, maternal mental health, pregnancy and child, postnatal and MCHAT score variables by screen exposure on survey-weighted data (%), ELFE cohort, France, 2011–2013

Variables	Tv/computer/tablet/smartphone use (*N* = 12 950)				
*Family socio-demographic characteristics*	Rarely/never (1714)	Monthly (*N* = 521)	Weekly *N* = 2517)	Daily (*N* = 8198)	*P*-value
**Maternal age: ≥ 30 years**	55.7%	59.6%	59.8%	49.8%	< .0001
**Paternal age: ≥ 30 years**	67.6%	73.4%	72.9%	71.1%	< .0001
**Mother born in France**	88.3%	89.8%	86.3%	80.8%	< .0001
**Father born in France**	87.3%	87.4%	86.8%	80.5%	< .0001
**Mother’s educational level**					< .0001
*No diploma*	6.1%	2.0%	6.8%	10.1%	
< *Bachelor’s degree*	38.9%	29.0%	30.3%	45.1%	
*Bachelor’s degree*	16.6%	18.4%	20.8%	19.9%	
≥ *Master’s degree*	38.4%	50.5%	42.0%	24.9%	
**Number of children in the household**					< .0001
*1 child*	42.5%	46.5%	40.0%	44.4%	
*2–3 children*	51.0%	50.5%	53.5%	48.4%	
≥ *4 children*	51.0%	50.5%	53.5%	48.4%	
**Maternal residential area**					< .0001
*Greater Paris Region*	19.9%	23.7%	20.6%	19.5%	
*Paris and suburbs*	19.4%	16.2%	15.2%	18.5%	
*North-West*	5.1%	3.6%	5.9%	7.9%	
*East*	7.7%	6.9%	7.8%	9.0%	
*West*	16.5%	16.9%	16.5%	13.3%	
*South-West*	8.4%	6.4%	9.7%	8.3%	
*Centre-East*	12.2%	16.0%	11.5%	10.9%	
*South-East*	10.8%	10.4%	12.8%	12.7%	
**Paternal residential area**					< .0001
*Greater Paris Region*	19.8%	23.5%	20.7%	19.4%	
*Paris Basin*	19.6%	16.3%	15.1%	18.5%	
*North-West*	5.1%	3.6%	5.9%	7.9%	
*East*	7.7%	6.9%	7.8%	9.0%	
*West*	16.5%	16.9%	16.8%	13.2%	
*South-West*	8.4%	6.4%	9.6%	8.4%	
*Centre-East*	12.2%	16.0%	11.5%	10.9%	
*South-East*	10.8%	10.4%	12.7%	12.6%	
**Household monthly income**					< .0001
< *1,423 euros*	9.1%	5.0%	8.1%	1.5%	
*1,423–1,996 euros*	7.4%	8.2%	6.7%	8.3%	
> *1,996 euros*	83.4%	86.8%	85.1%	76.7%	
**Maternal depression**	12.1%	10.8%	13.0%	15.3%	< .0001
*Pregnancy and child characteristics*					
**Child’s sex (female)**	48.2%	54.0%	47.8%	49.8%	< .0001
**Child’s age at interview**					< .0001
≤ *24 months*	19.4%	19.3%	18.9%	18.3%	
*25 months*	29.2%	34.7%	34.0%	33.6%	
*26 months*	25.4%	27.7%	28.2%	27.5%	
> *26 months*	25.9%	18.3%	19.7%	20.6%	
**Twin birth**	6.0%	3.3%	2.3%	3.1%	< .0001
**Season of birth**					< .0001
*Spring*	18.7%	25.1%	22.6%	22.0%	
*Summer*	31.1%	29.4%	27.8%	24.9%	
*Autumn*	25.0%	18.7%	24.9%	27.8%	
*Winter*	25.3%	26.8%	24.6%	25.3%	
**Premature birth (< 37 weeks of gestation)**	5.1%	7.0%	5.0%	5.2%	< .0001
**Birth weight for gestational age**					< .0001
*Small (*< *10 percentile)*	3.5%	3.7%	1.5%	2.2%	
*Average (10–90 percentile)*	93.6%	94.0%	95.3%	95.0%	
*Large (*> *90 percentile)*	2.8%	2.2%	3.1%	2.8%	
**Apgar score (good)**	98.4%	99.9%	99.1%	98.6%	< .0001
*Postnatal characteristics*					
**Childcare arrangements**					< .0001
*Parents or family member*	39.8%	28.1%	32.1%	46.5%	
*Centre-based care*	22.2%	24.8%	24.8%	18.1%	
*Nanny or professional*	38.0%	47.1%	43.1%	35.3%	
**Activities with the child**					< .0001
≤ *4 activities*	7.5%	83.7%	69.6%	7.1%	
*5–6 activities*	16.6%	15.1%	15.5%	16.6%	
*7–8 activities*	25.4%	25.4%	27.8%	28.0%	
≥ *9 activities*	50.4%	51.1%	49.7%	48.3%	
**Risk of ASD**					< .0001
*Low*	77.3%	77.9%	78.0%	75.2%	
*Intermediate*	21.4%	21.3%	21.3%	23.9%	
*High*	1.2%	0.8%	0.6%	0.9%	

In IPW-controlled analyses (Table 2), compared to never or seldom-users, children who used screen media weekly or daily had an increased likelihood of being at intermediate risk of neurodevelopmental difficulties (ORs between 1.05–1.07), but a decreased likelihood of being at high risk (ORs between 0.60–0.75). Compared to children who never watched TV daily, had an increased likelihood of intermediate level risk (OR associated with > 2 h per day 1.11, 95% CI 1.06–1.17), but a decreased likelihood of a high risk of neurodevelopmental difficulties (ORs associated with 30 mn to > 2 h between 0.33–0.60). Compared to never or seldom-users, children who used a computer/tablet ≤ 1 h/day had a decreased likelihood of being at intermediate risk or high risk of neurodevelopmental difficulties (ORs: 0.94, 95% CI 0.91–0.96 and 0.24, 95% CI 0.22–0.26, respectively); children who used a Tables 1 and 2 hours per week also had a low likelihood of being at high risk of neurodevelopmental difficulties (OR 0.83, 95% CI 0.70–0.98).

**Table 2:** Associations between screen use and risk of autism risk (M-CHAT score): Inverse probability weighted (IPW), survey weighted, multinomial logistic regression model: Odds Ratios (OR), 95% Confidence Interval (CI). ELFE cohort, France, 2011–2013

	**Intermediate risk** **of autism** **OR (95%CI)**	***P*-value**	**High risk of autism** **OR (95%CI)**	***P*-value**
**Overall screen media use**				
**TV/computer/tablet/smartphone use**				
Rarely/never (*n* = 1714)	*Reference*		*Reference*	
Monthly (*n* = 521)	1.03 (0.93—1.14)	.48	0.89 (0.72—1.10)	.26
Weekly (*n* = 2,517)	1.07 (1.02—1.12)	.006	0.60 (0.50—0.73)	.002
Daily (*n* = 8,198)	1.05 (1.02—1.08)	.003	0.75 (0.62—0.91)	.008
**Number of hours of screen media use**				
**TV/Computer/tablet (hrs/week)**				
Never (*n* = 2124)	*Reference*		*Reference*	
≤ 1 h (*n* = 1198)	0.96 (0.90- 1.02)	.15	0.72 (0.61—0.86)	.002
1–2 h (*n* = 1292)	1.03 (0.99- 1.08)	.13	0.09 (0.08—0.11)	< .001
> 2 h (*n* = 7884)	1.01 (0.99—1.03)	.31	0.61 (0.54—0.68)	< .001
**TV (hrs/day)**				
Never (*n* = 1845)	*Reference*		*Reference*	
≤ 30 min (*n* = 3632)	1.03 (0.99—1.08)	.10	0.79 (0.63—0.99)	.04
30 min-1 h (*n* = 3029)	1.00 (0.96—1.05)	.80	0.33 (0.27—0.41)	< .001
1–2 h (*n* = 2257)	1.02 (0.98—1.05)	.31	0.60 (0.49—0.74)	.002
≥ 2 h (*n* = 846)	1.11 (1.06—1.17)	.006	0.52 (0.37—0.73)	.002
**Computer/tablet use (hrs/week)**				
Never (*n* = 8485)	*Reference*		*Reference*	
≤ 1 h (*n* = 2150)	0.94 (0.91—0.96)	.004	0.24 (0.22—0.26)	< .001
1–2 h (*n* = 781)	1.02 (0.98—1.05)	.30	0.82 (0.70—0.98)	.03
> 2 h (*n* = 596)	0.94 (0.85—1.04)	.19	0.83 (0.64 – 1.07)	.13

## Discussion

In a nationally-representative study of 2-year old children, a majority were exposed to screen media daily. In IPW-controlled analyses, screen media use, mainly TV, was associated with an increased likelihood of intermediate level risk of neurodevelopmental difficulties, but a reduced likelihood of a high risk. Our study adds to a growing literature showing elevated levels of screen-based media use among young children and possible associations with development. However, several studies showed elevated levels of screen use among children at risk of autism spectrum disorder [6, 8] and our data temper the results of prior investigations. Our data suggest a complex relationship between screen-based activities and risk of neurodevelopmental difficulties, including autism risk.

We need to acknowledge several limitations. First, ELFE is based on parents’ voluntary participation and may have left out children vulnerable to neurodevelopmental difficulties [9]. Nevertheless, the sample is heterogeneous-enough to study environmental risk factors of neurodevelopment [12, 15] and our data were weighted to be nationally representative. Second, ELFE is a longitudinal cohort study, but the data we used are cross-sectional and we cannot rule out that children’s neurodevelopmental difficulties could influence their screen use. This may in part explain lower levels of exposure among children at high risk of neurodevelopmental difficulties. To address this issue, our statistical analyses controlled for a range of covariates which precede and predict children’s screen use as well as ASD symptoms, which is a way of taking into account common sources of variation. Future studies with prospective designs are warranted to assess the role of early screen use on children’s risk of neurodevelopmental difficulties, particularly those that are within the range of ASD. Third, we used a single M-CHAT measure, which is probably less specific than repeated assessments and it is unlikely that all children identified as having an intermediate or high risk will actually be diagnosed with ASD [10]. This results in the possibility of misclassification, particularly in the intermediate risk group, and calls for close monitoring of children’s later neurodevelopmental outcomes [11]. Fourth, ELFE study participants were born in 2011, and children’s levels of screen use have since increased, particularly during the COVID-19 pandemic [1]. This implies that prevalence levels of screen exposure at 2 years are probably currently be higher than we report. However, the associations we observe between children’s media screen use and risk of neurodevelopmental difficulties are likely to be valid in current circumstance or may have increased.

Our study also has strengths which we would like to highlight. First, ours is one of few studies to examine the early risk of neurodevelopmental difficulties in a community-based nationally representative sample of young children. Second, to our knowledge, this is the first study in a general population setting to include a measure of computer/tablet and smartphone use in early childhood and study the relationship with neurodevelopment. One of the hypotheses explaining this association is the effect of electromagnetic field exposure on children’s brain development [6]. Third, we used propensity scores and inverse-probability weights to control for selection biases and for a large number of child and family characteristics predating measures of screen media use that could confound the association of interest, ruling out a number of alternative explanations.

Overall, young children exposed to screen media may experience some neurodevelopmental delays, captured by the intermediate risk category on the M-CHAT. Our data suggest that TV use seems more strongly associated with children’s ASD risk than other types of screen media. This may reflect children’s greater passivity in front of a TV screen, which should be confirmed by other research, using quantitative as well as qualitative designs. This association may reflect reduced play time and physical activity, as well as more limited interactions with other children and adults [5]. Low levels of screen use among children at high risk of neurodevelopmental difficulties suggest limited interest or specific parental behaviours in some – rather highly educated – families which restrict children’s exposure [16]. There is suggestion that early environmental exposures such as screen media exposure could increase the experience of symptoms evocative of ASD risk, however this should be further investigated in studies which follow longitudinally large samples of children potentially at risk of ASD.

## Conclusion

Overall, screen media, mainly TV, use early on in life is related to children’s neurodevelopmental difficulties, but these associations appear to be complex.

## Data Availability

The datasets analysed during the current study are not publicly available due to regulatory restrictions in place in France but are available from the corresponding author on reasonable request.

## Abbreviations

OR: Odds Ratio
ASD: Autism-Spectrum Disorder
IPW: Inverse-Probability Weight
MI-FCS: Missing data Imputation -Fully Conditional Specification
